# Pharmacokinetics and the Dermal Absorption of Bromochlorophene, a Cosmetic Preservative Ingredient, in Rats

**DOI:** 10.3390/toxics10060329

**Published:** 2022-06-16

**Authors:** Yong-Jae Lee, Hyang-Yeon Kim, Quynh-Lien Pham, Jung-Dae Lee, Kyu-Bong Kim

**Affiliations:** 1 College of Pharmacy, Dankook University, 119 Dandae-ro, Cheonan 31116, Chungnam, South Korea; in0949@naver.com (Y.-J.L.); festivalkim@gmail.com (H.-Y.K.); nature.bn@gmail.com (Q.-L.P.); ljd0734@nate.com (J.-D.L.); 2 Center for Human Risk Assessment, Dankook University, Cheonan 31116, Chungnam, South Korea

**Keywords:** bromochlorophene, in vitro dermal absorption, in vivo pharmacokinetics

## Abstract

The cosmetic industry has flourished in recent years. Accordingly, the safety of cosmetic ingredients is increasing. Bromochlorophene (BCP) is a commonly used cosmetic preservative. To evaluate the effects of BCP exposure, in vitro dermal absorption and in vivo pharmacokinetic (PK) studies were conducted using gel and cream formulations. The Franz diffusion cell system and rat dorsal skin were used for tests according to the Korea Ministry of Food and Drug Safety guidelines for in vitro skin absorption methods. After the dermal application (1.13 mg/cm^2^) of BCP in the gel and cream formulations, liquid chromatography–mass spectrometry (LC–MS/MS) was used to evaluate the amount of BCP that remained unabsorbed on the skin (WASH), and that was present in the receptor fluid (RF), stratum corneum (SC), and (epi)dermis (SKIN). The total dermal absorption rate of BCP was 7.42 ± 0.74% for the gel formulation and 1.5 ± 0.9% for the cream formulation. Total recovery in an in vitro dermal absorption study was 109.12 ± 8.79% and 105.43 ± 11.07% for the gel and cream formulations, respectively. In vivo PK and dermal absorption studies of BCP were performed following the Organization for Economic Cooperation and Development guidelines 417 and 427, respectively. When intravenous (i.v.) pharmacokinetics was performed, BCP was dissolved in glycerol formal and injected into the tail vein (*n* = 3) of the rats at doses of 1 and 0.2 mg/kg. Dermal PK parameters were estimated by the application of the gel and cream formulations (2.34 mg/kg of BCP as an active ingredient) to the dorsal skin of the rats. Intravenous and dermal PK parameters were analyzed using a non-compartmental method. The dermal bioavailability of BCP was determined as 12.20 ± 2.63% and 4.65 ± 0.60% for the gel and cream formulations, respectively. The representative dermal absorption of BCP was evaluated to be 12.20 ± 2.63% based on the results of the in vivo PK study.

## 1. Introduction

The cosmetic industry has flourished in recent years. Cosmetics consist of various chemical compounds, such as surfactants, colorants, fragrances, and preservatives, in addition to active ingredients. Among these chemicals, preservatives are known to inhibit the growth of microbes and are essential for the safety of the product [1]. In Korea, 59 cosmetic preservatives have been listed for use [2]. Although usage doses have been stipulated for them, it is unclear whether these usage doses are safe. Therefore, appropriate risk-assessment studies are required.

Risk assessment is a process to identify the possible hazards of using specific products [3,4]. In the risk assessment of cosmetic ingredients, dermal absorption is considered an important factor in calculating the systemic exposure dosage (SED) of the application of the product [5]. By comparing the calculated SED and no observed adverse effect level (NOAEL) of the target compound, the margin of safety (MOS) can be measured (1).

MOS = NOAEL/SED,(1)

Bromochlorophene (BCP) is a preservative recommended for use at up to 0.1% composition in Korea. The logP_ow_ and solubility (in water) of BCP are 6.12 and <5 mg/L, respectively (Table S1). The toxicity of BCP has not been sufficiently reported. The rat oral LD_50_ has been reported to be 3700 mg/kg [6]. The Swedish Chemicals Agency (KEMI) reported that some preservatives with endocrine-disrupting properties, such as BCP, might pose a risk to animals when they enter the environment [7]. Moreover, the results of in silico and in vitro studies have shown that BCP interferes with nuclear receptor function and acts as an antagonist of the androgen receptor, estrogen receptor α, glucocorticoid receptor, and thyroid receptors α and β [8]. In addition, in a study by Won et al. on repeated-dose oral toxicity of BCP for 28 days, the relative weight of the kidneys in female rats increased at a dosage of 500 and 1000 mg/kg, and their hematological and histopathological characteristics were altered at these dosages [9]. They also reported in vivo dermal absorption in rats, but the analysis method used for the evaluation of BCP concentration and the formulation applied to the skin were unclear. Thus, to date, data on the dermal absorption of BCP are limited.

The objective of dermal absorption studies of cosmetic ingredients is to collect qualitative and/or quantitative information regarding the amount entering the systemic circulation of the human body [10]. In this study, in vitro and in vivo methods were used to determine the exact quantity of BCP absorbed by the skin. Franz diffusion cells were used to evaluate in vitro dermal absorption because they are simpler and cheaper to use compared with the application of flow-through methods. Consequently, they have the advantage of being able to expose a large surface area of the skin [11]; therefore, many researchers prefer to employ the Franz diffusion cell method [12,13]. However, the exact data obtained regarding skin absorption using this method remain controversial. In another study, dermal absorption was studied in vivo and its bioavailability was measured. Because in vivo dermal absorption involves application to the skin of live animals, more reliable data and pharmacokinetic information may be obtained through them. However, the differences between the results obtained with experimental animals can be more significant and more complex than those obtained following in vitro dermal studies [14].

Therefore, in the present study, we aimed to analyze the crucial dermal absorption of BCP through in vitro and in vivo pharmacokinetics (PK).

## 2. Materials and Methods

### 2.1. Chemicals

BCP (purity, 95%) and polyethylene glycol monooleyl ether (POE20) as the receptor fluid, glycerol as the vehicle, and felodipine (purity, >98%) as the internal standard (IS) were purchased from Tokyo Chemical Industry Co. (Chuo-ku, Tokyo, Japan). Phosphate buffered saline (PBS) was purchased from Sigma-Aldrich (St. Louis, MO, USA). Distilled water (D.W.) and acetonitrile (ACN) as the analysis solvents were obtained from Honeywell Burdick & Jackson Co. (St. Harvey, MI, USA). Formic acid was purchased from Merck Millipore (Kenilworth, NJ, USA).

### 2.2. Animals

Sprague Dawley (SD) rats (male, 8 weeks old, 228 ± 7 g) were supplied by Samtako Co. (Osan, Korea). The Institutional Animal Care and Use Committee of Dankook University (IACUC) approved this dermal absorption research for the limited ingredient BCP of cosmetics (approval number DKU-15-040). Animals were housed at a temperature of 23 ± 2 °C with a 12 h (h) light–dark cycle, and a relative humidity of 50 ± 10% in plastic cages with free access to a standard diet (Samtako Co., Osan, Korea) and tap water. For the in vitro study, 8-week-old rats were sacrificed using CO_2_ to obtain shaven full-thickness dorsal skin, which was stored at −20 °C. For in vivo study, at 24 h before treatment, rats were anesthetized by intramuscular injection of 1.25% avertin (St. Louis, MO, USA) and surgically cannulated with polyethylene tube (PE 60, 0.76 mm i.d., 1.22 mm o.d., Becton Dickinson, Franklin Lakes, NJ, USA) in the right jugular vein for blood sampling. The dorsal hair of the rats was shaved to 7 × 7 cm^2^ for dermal application of BCP using electronic hair removers (SM-129, JOAS, Namyangju, Korea).

### 2.3. Formulations

To perform the skin absorption of BCP in different cosmetic formulations, gel and cream formulations were prepared by Medikinetics (Pyeongtaek, South Korea). The ingredients of the formulations were listed in Table S2. The BCP in the gel formulation is dispersed in the hydrophilic phase (99% of water and glycerin); whereas the cream formulation is dispersed in the more hydrophobic phase. Each formulation contained 1% BCP for the in vivo and in vitro studies.

### 2.4. In Vitro Dermal Absorption

The in vitro dermal absorption study followed the guidelines of the Korea Ministry of Food and Drug Safety [15] with a Franz diffusion cell system consisting of a vision Microette auto-sampler, stirring drive, circulating water bath, stirring control, and autofill (Hanson, Chatsworth, CA, USA). The volume of the receptor fluid chamber was 12 mL and the temperature of the receptor fluid was maintained in a circulating water bath at 32 °C. Before skin application of BCP, stability of BCP in receptor fluid (6% POE20) was checked for 48 h at 32 °C. The skin, after hydrating with normal saline for 5 min, was then placed between the donor and receptor compartments of the diffusion cells. Trans epidermal water loss (TEWL) was estimated up to 24 h using Tewameter^®^TM300 (Courage and Khazaka, Cologne, Germany). Each formulation (~200 mg) was applied to 1.77 cm^2^ of the donor chamber.

After collecting the receptor fluid sample (RF) at 0, 1, 2, 4, 8, 12, and 24 h, unabsorbed BCP on the skin was wiped off with an alcohol swab (WASH). To obtain absorbed BCP in the stratum corneum (SC), tape strips were applied 15 times to the skin, and the skin was cut into eight pieces (SKIN). At the end of the experiment, WASH, SC, and SKIN samples were extracted using 6% POE20 and placed on Multi Tube Vortexer (VX2500, VWR, Radnor, PA, USA) for 10 min and sonicated for 1 h. Two hundred microliters of ACN containing IS (100 ng/mL) was added to 50 µL of each sample (WASH, SC, SKIN, and RF) and then mixed in Eppendorf tubes for 30 s. After centrifugation of the mixture at 13,000 rpm for 10 min, 150 µL of the supernatant was transferred to another Eppendorf tube and mixed with an equal volume of water. Then, a portion of the mixture (approximately 300 µL) was filtered using a 0.2 µm PTFE filter (ADVANTEC, Dublin, CA, USA) and samples (5 µL) were injected for liquid chromatography–mass spectrometry (LC–MS/MS) analysis.

### 2.5. In Vivo Pharmacokinetics

Intravenous (i.v.) PK of BCP was performed according to the Organization for Economic Cooperation and Development (OECD) guideline 417 [12]. BCP was dissolved in glycerol formal as a vehicle at concentrations of 1 and 0.2 mg/mL and was injected intravenously at a dose of 1 mL/kg via the tail vein. Blood samples (>0.2 mL) for plasma were collected by cannulated right jugular vein at 1, 2, 5, 10, and 30 min, and 1, 2, 4, 6, 12, 24, 36, and 48 h post-dose. Plasma samples were harvested by centrifugation at 13,000 rpm for 10 min and stored at −20 °C until analysis.

Dermal PK of BCP was performed according to the OECD guidelines 417 and 427 [16,17]. In this study, 234 mg/kg of the formulation (gel or cream containing 1% BCP) was applied to the dorsal skin (4 cm × 4 cm). After 12 h, the applied surface was wiped off with alcohol swabs to remove the remaining BCP. Blood samples (approximately 0.2 mL) were taken periodically by cannulated right jugular vein at 15 and 30 min, and 1, 2, 4, 8, 12, 24, 36, and 48 h post-dose. Plasma samples were harvested by centrifugation at 13,000 rpm for 10 min and stored at −20 °C until analysis.

To analyze the in vivo (dermal and i.v. application) samples, 50 µL of the plasma was used, and extraction and analysis methods were the same as in vitro samples.

### 2.6. LC–MS/MS Instruments and Conditions

To analyze BCP from the in vivo and in vitro studies, the LC–MS/MS manufactured by Shimadzu (LCMS-8050 set, Kyoto, Japan) was used. The LC–MS/MS system consisted of an auto-sampler (SIL-30AC), LC pump (LC-30AD-1 and -2), column oven (CTO-20AC), and a coupled electrospray ionization (ESI) detector (LCMS-8050). A Zorbax SB-C8 column (150 × 2.1 mm, i.d. 3.5 µm, Agilent, Santa Clara, CA, USA) with Security Guard Cartridges RP-1 (4 × 3.0 mm; Phenomenex, CA, USA) was used to separate each compound. The mobile phase was adjusted using 95% ACN in 0.1% formic acid. Flow rate, column oven temperature, and injection volume were 0.5 mL/min, 40 °C, and 5 L, respectively. The electrospray ionization (ESI) source was operated in the negative mode for BCP and in the positive mode for IS. All samples were observed in multiple reaction monitoring (MRM) modes. In the MRM mode, the dwell time was 100 ms per MRM channel. Gas flow, source temperature, and nebulizing gas flow were set at 10 L/min, 300 °C, and 3 L/min, respectively. The collision energy was 26 V for BCP and −14 V for IS. The analytical conditions are summarized in Table S3.

### 2.7. Analytical Method Validation

Validation of the BCP analysis method in the extracted solvent and plasma was performed using the calibration curve and quality control (QC) samples for linearity, selectivity, sensitivity, accuracy, and precision [18].

In a volumetric flask (20 mL), 10 mg of BCP was weighed and dissolved in 6% POE20. Working solutions of BCP were prepared to the concentrations of 10, 50, 100, 500, 1000 and 2000 ng/mL. All the calibration samples were diluted 1/10 fold with each blank sample and prepared to 1, 5, 10, 50, 100, and 200 ng/mL concentrations. Similarly, QC samples were prepared to the concentrations of 1, 3, 15, and 150 ng/mL as the lower limit of quantification (LLOQ), low QC (LOQ), medium QC (MOQ), and high QC (HOQ) samples, respectively. Felodipine, used as an internal standard (IS), was diluted with ACN to a concentration of 100 ng/mL. To analyze BCP, each sample was subjected to in vitro and in vivo sample preparation.

Sensitivity and selectivity were evaluated by analyzing blank and LLOQ samples of WASH, SC, SKIN, RF, and plasma. The accuracy and precision were determined using three replicates of LLOQ, LOQ, MOQ, and HOQ in the same day (intra-day) or three consecutive days (inter-day) [18].

### 2.8. Data Analysis

In vitro dermal absorption was calculated using quantitative results of BCP from LC–MS/MS analysis of each sample as follows: skin absorption (%) = 100 (SKIN + RF)/(SK + RF + SC + WASH) [19]. In the in vivo study, the terminal elimination half-life (T_1/2_) was calculated to be 0.693/λ_z_. The slope of the last phase (λ_z_) is the individual estimate of the terminal elimination rate constant, which is calculated using log-linear regression of the terminal portions of the plasma concentration–time curves. The area under the plasma concentration–time curve from time zero to the last observation time point (AUC_all_) was calculated using the trapezoidal rule, and the AUC to infinity time (AUC_inf_) was obtained by adding C_n_/λ_z_ to the AUC_all_ (C_n_: last concentration of chemical). Systemic clearance (CL) was calculated as dose/AUC. The apparent volume of the distribution (V_d_) during the terminal phase was calculated as CL/λ_z_. The peak plasma concentration (C_max_) and time to reach C_max_ (T_max_) were determined directly from these observations. The relative bioavailability (F) after dermal application was calculated as F (%) = 100 (Dose_iv_·AUC_dermal_)/(Dose_dermal_·AUC_iv_) [16,20].

The plasma concentration–time data were analyzed using a non-compartmental WinNonlin model (version 2.1, Pharsight, NC, USA) to acquire i.v. and dermal pharmacokinetic parameters.

## 3. Results

### 3.1. Analytical Method Validation

The qualitative/quantitative BCP analytical method was validated for the in vitro and in vivo studies by evaluating its stability, accuracy, selectivity, and precision. Mass chromatograms obtained for the WASH, SC, SKIN, RF, and plasma samples at the LLOQ levels are shown in Figure S1. Under the analytical conditions described above, BCP and IS were eluted at 0.91 and 0.97 min, respectively, and matrix effects from endogenous interferences were not observed for the five different samples.

The linearity of the calibration curves was adjusted for five different samples in the range of 1–200 ng/mL, and the linear regression (r^2^) of these samples were greater than 0.993 (Table 1). For the QC samples (WASH, SC, SKIN, RF, and plasma), the accuracy and precision obtained were 86.90–108.03% and 1.22–10.66% for intra-day and 93.17–110.00% and 0.58–8.04% for inter-day, respectively (Table 2).

### 3.2. In Vitro Dermal Absorption

**Figure 1:**
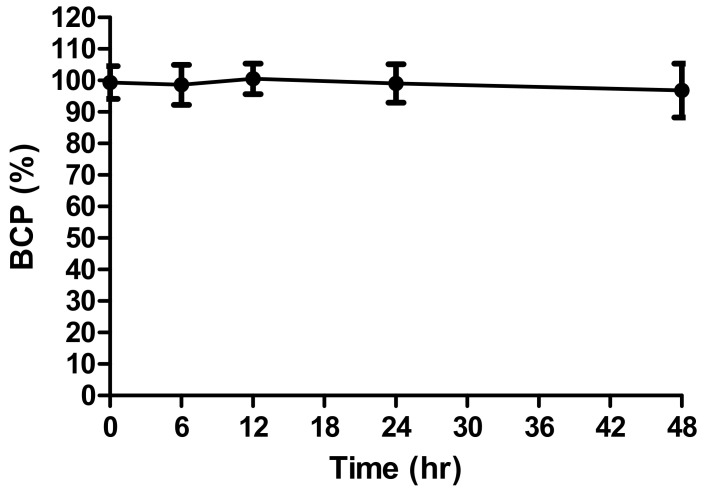
The stability of bromochlorophene dissolved in 6% POE20 at 0.5 mg/mL in sink conditions (*n* = 3).

In the in vitro skin absorption system, it is important to stabilize the target compound in the receptor fluid to obtain exact skin absorption. In this study, BCP was stable in 6% POE20 for 48 h (Figure 1), making it suitable for BCP for the in vitro skin absorption study.

The results of the in vitro dermal absorption study are summarized in Table 3. The total recoveries of BCP for the in vitro dermal absorption test were 109.12 ± 9.79% for the gel formulation and 105.43 ± 11.07% for the cream formulation. Total dermal absorption (SKIN + RF) of BCP was higher for the gel formulation than the cream formulation (7.43 ± 0.74% for the gel formulation and 1.48 ± 0.93% for the cream formulation). The percentage of BCP in receptor fluid after 24 h was 0.0017 ± 0.0002% and 0.0012 ± 0.001% for the gel and cream formulations, respectively.

### 3.3. Pharmacokinetics

**Figure 2:**
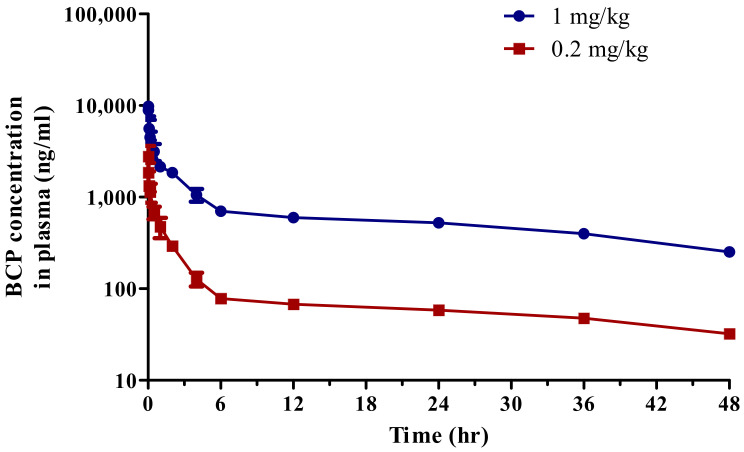
Average plasma concentration–time profiles of BCP after intravenous injection at a dose of 1 (blue) and 0.2 (red) mg/kg to rat (*n* = 3).

The developed analytical method was used to determine BCP pharmacokinetics in rats. The average plasma concentration–time profiles after i.v. injection of dissolved BCP at doses of 0.2 and 1 mg/kg are shown in Figure 2. The pharmacokinetic parameters of the intravenous injection are summarized in Table 4. The T_1/2_s of 0.2 and 1 mg/kg were comparable at 34.48 ± 5.87 h for 0.2 mg/kg and 30.52 ± 1.83 h for 1 mg/kg. V_d_s were 1742.43 ± 71.97 L/kg for 0.2 mg/kg and 1058.30 ± 50.17 L/kg for 1 mg/kg. CLs were 34.58 ± 4.84 for 0.2 mg/kg and 24.05 ± 0.54 for 1 mg/kg. The AUC_all_, AUC_inf_, and C_max_ were estimated in a dose-dependent manner. The AUC_all_s were 4190.78 ± 319.68 ng·h/mL for 0.2 mg/kg and 30,458.03 ± 366.90 ng·h/mL for 1 mg/kg. AUC_inf_s were 5855.42 ± 766.69 ng·h/mL for 0.2 mg/kg and 41,591.66 ± 939.34 ng·h/mL for 1 mg/kg. C_max_s were 4192.57 ± 1685.90 ng/mL for 0.2 mg/kg and 11,385.42 ± 1526.38 ng/mL for 1 mg/kg.

**Figure 3:**
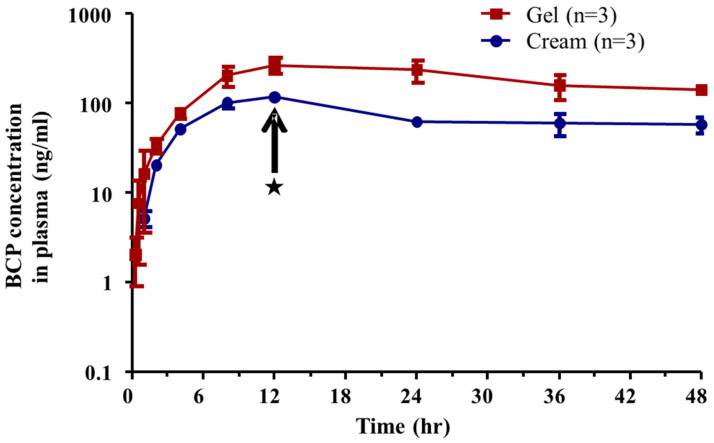
Average plasma concentration–time profiles of BCP after dermal application in gel (red) and cream (blue) formulation at 234 mg/kg (BCP of 2.34 mg/kg) (*n* = 3). ★ is the time of swabbing unabsorbed BCP on the skin surface.

The average plasma concentration–time profiles of BCP after dermal application of the gel and cream formulation at a dose of 234 mg/kg (2.34 mg /kg as a BCP; 3 mg/cm^2^ as an application of formulation) are shown in Figure 3. When the applied area of the skin surface was wiped off at 12 h by swabbing with alcohol cotton, the elimination period of BCP in the plasma was observed for 12–48 h. The pharmacokinetic parameters of the dermal applications are summarized in Table 5. Regardless of the formulation type, T_max_ was the same for both formulations. The T_1/2_ was comparable to that of the gel and cream formulations. However, T_1/2_ was higher in the dermal application than in the i.v. injection. T_1/2_ was 38.54 ± 9.54 h for gel and 39.41 ± 11.70 h for cream. C_max_ was higher in the gel formulation than in the cream formulation. C_max_ was 259.77 ± 50.03 ng/mL for the gel formulation and 116.19 ± 10.38 ng/mL for the cream formulation. The AUC_all_ and AUC_inf_ were higher in the gel formulation than in the cream formulation. The AUC_all_ was 8687.81 ± 1843.71 ng·h/mL for the gel formulation and 3309.16 ± 403.33 ng·h/mL for the cream formulation. AUC_inf_ was 16,356.04 ± 2518.43 ngh/mL for the gel formulation and 6708.17 ± 2149.84 ng·h/mL for the cream formulation. Finally, the dermal bioavailability of BCP was higher in the gel formulation (12.20 ± 2.63%) than in the cream formulation (4.65 ± 0.60%).

## 4. Discussion

BCP is a preservative ingredient with a 0.1% concentration limit for cosmetics in Europe and Korea. Although that limit is safe for cosmetics, the risk assessment (calculating SED and MOS) of BCP as a cosmetic preservative has not been reported. To evaluate the risk of BCP, there is a need to study its dermal absorption. The dermal absorption rate of cosmetic ingredients is an important factor for risk assessment, owing to its effect on systemic exposure and toxicity [21]. In the present study, dermal absorption of BCP was assessed using an in vitro Franz diffusion cell system and in vivo dermal bioavailability analysis.

Prior to the in vitro and in vivo dermal absorption studies, the LC–MS/MS analysis method for BCP was validated. According to the guidelines in Korea and USA [18,22], the accuracy and precision ranged between 80 and 120% for LLOQ and QC samples at low, medium, and high concentrations ranged between 85 and 115%. In total, the accuracy and precision of the QC samples for all concentrations of the five different samples were acceptable according to the Ministry of Food and Drug Safety (MFDS) and Food and Drug Administration (FDA) guidelines (Table 2).

In the skin, the physicochemical properties or formulation type of the cosmetic ingredients influence dermal absorption [20,23,24,25,26,27,28,29,30,31,32]. According to the results of in vitro dermal absorption analysis, the gel formulation (7.43 ± 0.74%) was more absorbed than the cream formulation (1.48 ± 0.93%) (Table 3). In this study, the in vitro dermal test was performed in an occlusion state, which blocked moisture evaporation [33]. Physical characteristics of the corneocytes stacked on the stratum corneum are maintained until the stratum corneum is changed by an enhancer (such as sulfoxides, water, pyrrolidones, glycols, alcohols, and azones) or skin diseases [23,24]. If the skin is continuously exposed to water (in occlusion conditions), which is known as a dermal absorption enhancer, the corneocytes are filled with water, leading to their expansion. When swelling stratum corneum by water in occlusion conditions, the physical structures of the epidermis and dermis were maintained. Thus, only the stratum corneum changed its physical structure [34]. Moreover, the intercellular space of the skin is the main absorption route for materials that are absorbed through the stratum corneum. The intercellular space is composed of various lipid molecules (sterol esters, cholesterol, ceramides, fatty acids, and cholesteryl sulfate). Thus, hydrophilic materials are less absorbed by normal skin because the intercellular spaces contain many hydrophobic chains [25,26]. At the same time, lipophilic substances in the hydrophilic formulation (emulsion gel) were more permeated to stratum corneum than those in the lipophilic formulation (petroleum) [30]. In addition, the accumulation rates of homosalate (LogP_ow_ = 5.94), ethylhexyl methoxycinnamate (LogP_ow_ = 5.96), benzophenone-3 (LogP_ow_ = 3.58), butyl, ethylhexyl salicylate (LogP_ow_ = 6.02), and methoxydibenzoyl methane (LogP_ow_ = 4.68) in stratum corneum were higher for the gel formulation than for vaseline [20,30]. Based on this characteristic, lipophilic compounds are absorbed more in the hydrophilic formulation because of its lipophilic properties. Similarly, this phenomenon was reported not only in the stratum corneum but also in the (epi)dermis. Im et al. reported that testosterone has a LogP_ow_ of 3.3 and was more absorbed in solution than the cream formulation [35]. For these reasons, BCP (LogP_ow_ = 6.12) in the gel formulation might show a higher absorption percentage compared to BCP in cream formulations of in vitro and in vivo studies.

In the in vivo dermal study, 2.34 mg/kg of BCP was applied to rat dorsal skin and 12.2 ± 2.63% of bioavailability was observed. However, in another in vivo dermal study [9], BCP absorption in the hydrogel formulation was 29.25 ± 6.09% following 0.262 mg/kg rat dorsal application. In accordance with Im et al., the application doses affected skin absorption. At 5 mg/cm^2^ or 5 µL/cm^2^, caffeine and testosterone showed a higher absorption % than 50 mg/cm^2^ or µL/cm^2^ [35]. Because their applied concentration (0.262 mg/kg) was lower than that used in this study (2.34 mg/kg), they might have a higher reported % absorption. After the in vivo dermal application of the gel and cream formulation of BCP, continuous absorption was allowed up to 12 h, after which the applied area was wiped off with alcohol swabs to remove the unabsorbed BCP. Moreover, both formulations similarly reached steady state within 12 h and had the same T_max_ (12 h). After wiping off the unabsorbed BCP, the plasma concentration of BCP decreased constantly over 48 h. According to the in vitro study, most of BCP was retained in the SKIN sample (Table 3). When BCP is in the elimination period, it is considered that BCP in (epi)dermis might be slowly penetrated due to its strong affinity to the lipophilic components of the skin layers. Although levels of permeated BCP differed between the gel and cream formulations, there were no differences in T_1/2_. Moreover, the T_1/2_s of BCP after i.v. injection were almost same as those of in vivo dermal absorption (Table 4 and Table 5). Since T_1/2_ is an intrinsic property of a compound, it seems to be not changed easily.

In the i.v. injection study, AUCs and C_max_ increased in a dose-dependent manner (Table 4). However, BCP T_1/2_, V_d,_ and CL at 1 mg/kg were lower than those observed following the 0.2 mg/mL application. T_1/2_, CL, and V_d_ are dependent variables (t_1/2_ = 0.693 × V_d_/CL). Clearance was calculated by the amount of compound eliminated and the average concentration of the target compound in the plasma (CL= A/P, where A is the amount of compound eliminated from the plasma and P is the average plasma concentration). Since the AUCs (AUC_all_ and AUC_inf_) of BCP in the plasma were approximately 7-fold higher at 1 mg/kg, it is possible to have a low CL rate in a higher-dose application [36,37,38].

Many studies have reported a good correlation between in vitro and in vivo dermal absorption [32,39,40,41]. In the present study, BCP was absorbed more through the gel formulation than through the cream formulation in both the in vivo and in vitro studies. Because in vivo studies use a physiologically and metabolically intact system, the in vivo dermal absorption test or dermal PK is mainly used when a more precise assessment is needed [17,42]. Therefore, the total dermal absorption of BCP can be used to determine the relative dermal bioavailability in rats. Based on these results, dermal absorption of BCP was determined to be 12.20 ± 2.63% through the in vivo dermal study.

## 5. Conclusions

A specific and sensitive analytical method for BCP using LC–MS/MS was developed and validated in various matrices. An analytical method was applied to determine BCP concentration from rat plasma after i.v. injection and dermal application of BCP to calculate relative bioavailability using i.v. and dermal PK. Both the in vitro and in vivo studies showed that BCP was better dermally absorbed in the gel formulation than in the cream formulation. Based on in vitro dermal absorption and in vivo pharmacokinetic results, total dermal absorption of BCP was determined to be 12.20 ± 2.63%. This dermal absorption rate of BCP might be used to estimate the systemic exposure dosage (SED) of BCP for further study of risk (or safety) assessment.

## Figures and Tables

**Table 1:** Linearities of BCP calibration sample in various matrices.

Compound	Matrix	Calibration Sample (ng/mL)	Linearity (r^2^)
Bromochlorophene	WASH	1, 5, 10, 50, 100, 200	0.9994
	SC		0.9996
	SKIN		0.9994
	RF		0.9993
	Plasma		0.9995

**Table 2:** Accuracy and precision of BCP QC sample in various matrices (*n* = 3).

Compound	Conc. (ng/mL)	Intra-Day (%)		Inter-Day (%)	
		Accuracy	Precision	Accuracy	Precision
WASH	1	91.70	2.93	99.30	4.78
	3	100.56	4.46	97.50	4.23
	15	99.54	1.52	101.08	3.34
	150	101.77	2.04	100.68	2.43
S.C	1	86.90	4.26	110.00	0.79
	3	101.36	2.36	104.11	1.43
	15	100.63	4.33	98.30	0.58
	150	96.86	1.84	98.24	1.72
SKIN	1	94.47	4.39	104.4	8.05
	3	108.03	4.44	101.18	5.65
	15	103.62	1.29	106.05	0.96
	150	101.08	1.22	101.88	4.17
R.F	1	102.4	10.66	93.17	4.29
	3	95.94	4.60	102.87	8.59
	15	102.42	2.05	101.73	2.36
	150	104.75	2.47	96.79	3.63
Plasma	1	107.70	4.93	104.5	8.04
	3	100.68	6.35	107.61	4.31
	15	107.56	4.28	104.79	2.32
	150	105.04	5.11	102.18	3.22

**Table 3:** In vitro dermal absorptions of 1% BCP in gel and cream formulations (*n* = 3).

Matrix	Formulation	
	Gel (%)	Cream (%)
WASH	95.56 ± 10.86	101.88 ± 9.62
S.C	4.49 ± 1.52	0.89 ± 0.15
SKIN	7.42 ± 0.74	1.48 ± 0.93
R.F	0.0017 ± 0.74	0.0012 ± 0.001
Total absorption (SKIN + RF)	7.43 ± 0.74	1.48 ± 0.93
Recovery	109.12 ± 8.79	105.43 ± 11.07

**Table 4:** Pharmacokinetic parameters of BCP following i.v. injection at doses of 1 and 0.2 mg/kg to rats (*n* = 3).

Parameter	Dose of Administration	
	0.2 mg/kg	1 mg/kg
T_1/2_ (h)	34.48 ± 5.87	30.52 ± 1.83
C_max_ (ng/mL)	4192.57 ± 1685.90	11,385.42 ± 1526.38
AUC_all_ (ng·h/mL)	4190.78 ± 319.68	30,458.03 ± 366.90
AUC_inf_ (ng·h/mL)	5855.42 ± 766.69	41,591.66 ± 939.34
V_d_ (L/kg)	1742.43 ± 71.97	1058.30 ± 50.17
CL (mL/min/kg)	34.58 ± 4.84	24.05 ± 0.54

T_1/2_ (h), terminal elimination half-life; C_max_ (ng/mL); peak plasma concentration; AUC_all_ (ng·h/mL), area under the curve from zero to the last observation time point; AUC_inf_ (ng·h/mL), area under the curve from zero to infinity time; V_d_ (L/kg), volume of distribution; CL (mL/min/kg), systemic clearance. Each value represents the mean ± S.D.

**Table 5:** Pharmacokinetic parameters of BCP following dermal application at a dose of 234 mg/kg (BCP of 2.34 mg/kg) to rats (*n* = 3).

Parameter	Dose of Administration	
	Gel	Cream
T_1/2_ (h)	38.54 ± 9.54	39.41 ± 11.70
T_max_ (h)	12.00 ± 0.00	12.00 ± 0.00
C_max_ (ng/mL)	259.77 ± 50.03	116.19 ± 10.38
AUC_all_ (ng·h/mL)	8687.81 ± 1843.71	3309.16 ± 403.33
AUC_inf_ (ng·h/mL)	16,356.04 ± 2518.43	6708.17 ± 2149.84
F (%)	12.20 ± 2.63	4.65 ± 0.60

T_1/2_ (h), terminal elimination half-life; T_max_ (h), time to reach the peak plasma concentration; C_max_ (ng/mL), peak plasma concentration; AUC_all_ (ng·h/mL), area under the curve from zero to the last observation time point; AUC_inf_ (ng·h/mL); area under the curve from zero to infinity time; F (%), dermal bioavailability. Each value represents the mean ± S.D.

## Data Availability

Not applicable.

## Supplementary Materials

The following supporting information can be downloaded at: https://www.mdpi.com/article/10.3390/toxics10060329/s1, Figure S1. Representative MRM chromatograms of BCP and IS at LLOQ in WASH, SC, SKIN, RF, and plasma. Table S1: Physicochemical properties of BCP. Table S2: Contents of the gel and cream formulations containing 1% BCP. Table S3: Analytical conditions of BCP and IS of LC–MS/MS.
