# Supplementary material for: Pharmacokinetics and the Dermal Absorption of Bromochlorophene, a Cosmetic Preservative Ingredient, in Rats
Source: Toxics. 2022 Jun 16;10(6):329. doi: 10.3390/toxics10060329 (PMC9229563; doi:10.3390/toxics10060329)
Supplement: Supplementary file 1 [file toxics-10-00329-s001.zip › toxics-1745491-supplementary.pdf]

toxics

# Supplementary Materials: Pharmacokinetics and the Dermal Absorption of Bromochlorophene, A Cosmetic Preservative Ingredient, in Rats

Yong- Jae Lee, Hyang Yeon Kim, Quynh Lien Pham, Jung Dae Lee and Kyu-Bong Kim

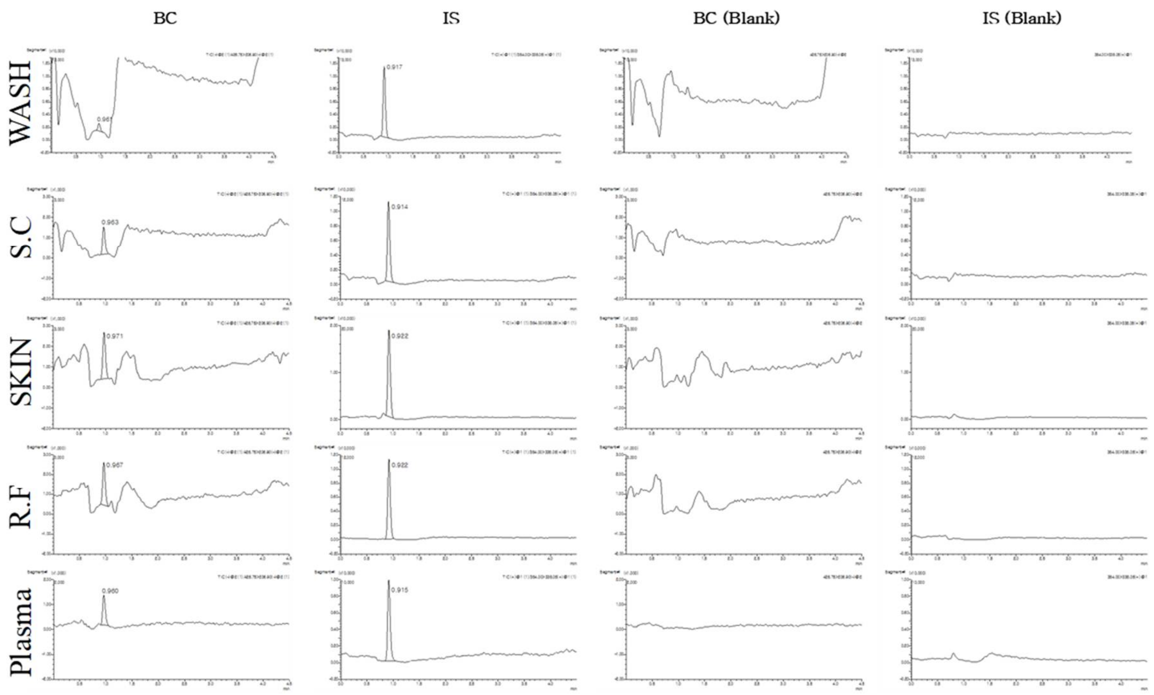


**Figure S1.** Representative MRM chromatograms of BCP and IS at LLOQ in WASH, SC, SKIN, RF, and plasma.

**Table S1.** Physicochemical properties of BCP.

| | | |
|---|---|---|
| Structure of chemical | 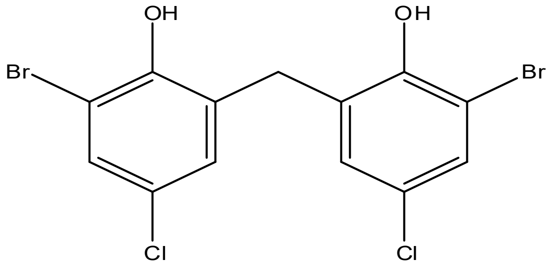  | |
| INCI name | Bromochlorophene | |
| EINECS name | 2,2'-Methylenbis (6-bromo-4-chlorophenol) | |
| CAS number | 15435-29-7 | |
| EC number | 239-446-8 | |
| Molecular formula | $C_{13}H_8Br_2Cl_2O_2$ | |
| Molar mass | 426.92 | |
| Solubility in water | < 5 mg/L | |
| Log $P_{ow}$ | 6.12 | |
| Effect of preservative | gram-positive bacteria | good |
| | gram-negative bacteria | moderate |
| | yeasts / moulds | inadequate |
| | head-space pretection | no |

**Table S2.** Contents of gel and cream formulations containing 1 % BCP.

| Content | Gel (%,w/w) | Cream (%,w/w) |
|---|---|---|
| Distilled water | 75.4 | 54 |
| Glycerin | 23.6 | 4 |
| Grape seed oil | | 28 |
| Benzyl alcohol | | 5 |
| Olive glutinate | | 3 |
| Hydroxypropyl cyclodextrin | | 5 |
| Bromochlorophene | 1 | 1 |
| Total | 100 | 100 |

**Table S3.** Analytical conditions of BCP and IS of LC-MS/MS.

| | **Bromochlorophene** | **Felodipine (IS)** |
|---|---|---|
| Instrument | | |
| HPLC | LC-30AD-1 and -2, SIL-30AC, CTO-20AC (Shimadzu, Kyoto, Japan) | |
| MS/MS | LCMS-8050 (Shimadzu, Kyoto, Japan) | |
| Column | Zorbax SB-C8 (150 × 2.1 mm, i.d. 3.5 µm , Agilent, USA) | |
| Guard column | Security Guard Cartridges RP-1 (4 × 3.0 mm; Phenomenex, CA, USA) | |
| Chromatographic condition | | |
| Mobile phase | 95% ACN in 0.1% formic acid | |
| Column oventemperature (°C) | 40 | |
| Autosampler temperature (°C) | 10 | |
| Flow rate (mL/min) | 0.5 | |
| Running time (min) | 4.5 | |
| Injection volume (mL) | 5 | |
| MSMS condition | | |
| Ionization mode | Electro spray ionization, multi reaction monitoring | |
| Polarity | Negative | Positive |
| Mass transition (m/z) | 426.75 → 206.90 | 384.00 → 338.05 |
| Dwell time (ms) | 100 | 100 |
| Q1 pre vias | 19 | -13 |
| Q3 pre vias | 21 | -25 |
| Collision energy (V) | 26 | -14 |
| Gas flow (L/min) | 10 | |
| Neubulizing gas flow (L/min) | 3 | |
| Source temperature (°C) | 300 | |
